# A Novel Method of the Generalized Interval-Valued Fuzzy Rough Approximation Operators

**DOI:** 10.1155/2014/783940

**Published:** 2014-08-04

**Authors:** Tianyu Xue, Zhan'ao Xue, Huiru Cheng, Jie Liu, Tailong Zhu

**Affiliations:** ^1^College of Computer and Information Engineering, Henan Normal University, Xinxiang 453007, China; ^2^College of Mathematics and Information Science, Henan Normal University, Xinxiang 453007, China

## Abstract

Rough set theory is a suitable tool for dealing with the imprecision, uncertainty, incompleteness, and vagueness of knowledge. In this paper, new lower and upper approximation operators for generalized fuzzy rough sets are constructed, and their definitions are expanded to the interval-valued environment. Furthermore, the properties of this type of rough sets are analyzed. These operators are shown to be equivalent to the generalized interval fuzzy rough approximation operators introduced by Dubois, which are determined by any interval-valued fuzzy binary relation expressed in a generalized approximation space. Main properties of these operators are discussed under different interval-valued fuzzy binary relations, and the illustrative examples are given to demonstrate the main features of the proposed operators.

## 1. Introduction

Rough set theory proposed by Pawlak [1] is an extension of set theory for the study of intelligent systems characterized by inexact, uncertain, or insufficient information. The core of the rough set theory and its applications is to define a pair of lower and upper approximation operators, and an equivalence relation is a key and primitive notion in Pawlak's rough set model [1]. This equivalence relation is the key concept of Pawlak's rough set model, but also a very strict condition, which may limit the applicability of the rough set model [2, 3]. To solve this problem, several authors have generalized the notion of the approximation operators by using nonequivalent binary relations. The most important research is the amalgamation of fuzzy set theory and rough set theory [2, 4–7] as well as the rough set theory based on generalized binary relations [8–13]. Pawlak first discussed the relation between rough sets and fuzzy sets in [6]. Dubois and Prade [4] proposed the fuzzy rough set theory by amalgamating the fuzzy set theory with the rough set theory. In addition, based on the definition of neighborhood operators, Yao [8–11] studied the rough set theory based on the generalized binary relation, that is, the generalized rough set theory. Recently, Wu et al. [14–19] defined the generalized fuzzy rough set theory based on the study of the fuzzy rough set theory and the generalized rough set theory, and Zhu [12] studied generalized rough sets based on relations.

**Figure 1:**
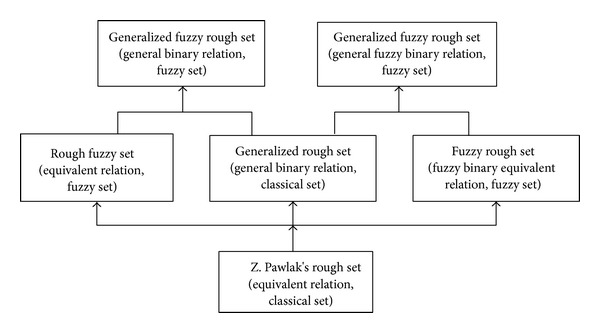
Select dependencies in rough set theory.

A rough set model is composed of two parts: the approximation space and the approximated object. Rough set theory comes with a lot of extensions and generalizations. Yao et al. researched the generalized rough sets by considering sets and relations of the approximation space and the approximated object [9, 16]. In Pawlak's rough set model [6], the relation of approximation spaces is a classical binary equivalence relation and the approximated object is a set. If the equivalence relation is weakened to a general binary relation, the equivalence relation is a special case of the general binary relation. The set theory is generalized to the form of the fuzzy set theory, so that the classical set theory is a special case of the fuzzy set theory. These relationships are outlined in Figure 1.

Most researches on the fuzzy rough set theory focus on point-valued fuzzy sets and point-valued fuzzy binary relations. But the fuzzy notion described by using point values may lose some available information in the real-life information systems sometimes. If the description is done by interval values, it may acquire a better effectiveness than that by using point ones, for example, a self-evolving interval type-2 fuzzy neural network with online structure and parameter learning [20], encoding words into interval type-2 fuzzy sets using an interval approach [21], and corrections to aggregation using the linguistic weighted average and interval type-2 fuzzy sets [22]. Gong et al. [23] proposed a kind of interval-valued rough fuzzy set model based on an equivalent relation and applied the model to acquire rules from the interval-valued fuzzy information systems. It is very significant to apply the interval-valued fuzzy set in researching the rough set theory. Yeung et al. [24] generalized the fuzzy rough sets by means of arbitrary fuzzy relations and presented a general framework for the study of fuzzy rough sets by using both constructive and axiomatic approaches. Wu et al. [25] generalized the concept of fuzzy rough sets to interval type-2 fuzzy environments and proposed a method of attribute reduction within the interval type-2 fuzzy rough set framework. Xue et al. [26] generalized interval-valued fuzzy rough approximation operators. Zhang et al. [27] studied the characterization of generalized interval-valued fuzzy rough sets on two universes of discourse. The positive approximation and converse approximation in interval-valued fuzzy rough sets have been studied in [28]. Zhang and Jiang [29] proposed a note on interval-valued fuzzy rough sets and interval-valued intuitionistic fuzzy sets. Zhang et al. [30] proposed a general frame for intuitionistic fuzzy rough sets. Xu et al. [31] studied an axiomatic approach of interval-valued intuitionistic fuzzy rough sets based on interval-valued intuitionistic fuzzy approximation operators. Zhang and Tian [32] studied interval-valued intuitionistic fuzzy rough sets based on implicators. Wu and Zhou [33] studied intuitionistic fuzzy topologies based on intuitionistic fuzzy reflexive and transitive relations. Zhang et al. [34] proposed a variable-precision-dominance-based rough set approach to interval-valued information systems. Liang and Liu [35] studied three-way decisions with interval-valued decision-theoretic rough sets. Dai et al. [36] proposed an uncertainty measurement for interval-valued decision systems based on extended conditional entropy. Zhang et al. [37] studied multiconfidence rule acquisition and confidence-preserved attribute reduction in interval-valued decision systems. Ma and Hu [38] studied topological and lattice structures of L-fuzzy rough sets determined by lower and upper sets. Hao and Li [39] discussed the relationship between L-fuzzy rough set and L-topology. Zhang et al. [40] studied the union and intersection operations of rough sets based on various approximation spaces. She and He [41–43] studied rough approximation operators on R0-algebras (nilpotent minimum algebras) with an application in formal logic L, the rough consistency measures of logic theories, and approximate reasoning in rough logic and the structure of the multigranulation rough set model as well. Yang et al. [44] studied the combination of interval-valued fuzzy set and soft set. In terms of these researches above, a number of important conclusions are drawn, which exhibit great significance to research the rough fuzzy set theory. However the generalized interval-valued fuzzy rough set theory under the generalized relations needs to be further investigated.

In this paper, we further study the generalized fuzzy rough approximation operators defined in [16]. In particular, from the viewpoint of constructive approach, we reconstruct the lower approximation operator on the premise of the fact that the upper approximation operator is not changed and expand it to interval environments. It is proved that the lower approximation operator is equivalent to the generalized interval Dubois fuzzy rough approximation operator in the approximation space formed by arbitrary binary interval-valued fuzzy relations. Also, properties of the operators are discussed under the different binary interval-valued fuzzy relations.

The rest of the paper is organized as follows. In Section 2, we give some basic notions of interval-valued fuzzy sets and interval-valued fuzzy relations. In Section 3, we study the generalized fuzzy rough approximation operators defined in [16]. In Section 4, from the viewpoint of constructive and interval approach, we reconstruct new lower and upper approximation operators of the generalized interval-valued fuzzy rough sets. In Section 5, we prove some properties of the generalized interval-valued fuzzy rough approximation operators and the presented scheme by the extensive analysis results. In Section 6, we bring forward some conclusions and highlight further work.

## 2. Basic Concepts of Interval-Valued Fuzzy Sets and Interval-Valued Fuzzy Relations

In this section, we introduce some basic notions and properties related to interval-valued fuzzy sets which will be used in this paper. We first review an interval-valued subset originated by [28]. We first review some basic concepts.

Let *I* be a closed unit interval; that is, *I* = [0,1]. [*I*] = {[*a* ^−^, *a* ^+^] : *a* ^−^ ≤ *a* ^+^, *a* ^−^, *a* ^+^ ∈ *I*} is the set of all interval-valued subsets of *I*. *a* = [*a* ^−^, *a* ^+^]∈[*I*] is an interval value. When *a* ^−^ = *a* ^+^, the interval-valued *a* = [*a* ^−^, *a* ^+^] becomes a real number in [*I*]. In particular, real numbers return intervals of zero length, say 1 = [1,1] and 0 = [0,0].

**Definition 1 .**

Let *a*, *b* ∈ [*I*]. *a* ≤ *b* if and only if *a* ^−^ ≤ *b* ^−^, *a* ^+^ ≤ *b* ^+^; *a* = *b* if and only if *a* ^−^ = *b* ^−^, *a* ^+^ = *b* ^+^; *a* < *b* if and only if *a* ≤ *b* and *a* ≠ *b*.

**Definition 2 .**

Let *a*, *b* ∈ [*I*]. *a*≰*b* indicates that *a* is not less than or equal to *b*; *a* ≮ *b* indicates that *a* is not less than *b*; *a*≱*b* indicates that *a* is not greater than or equal to *b*; *a*≯*b* indicates that *a* is not greater than *b*.

According to the order relation defined in Definition 1, different elements in [*I*] may not exhibit order relations, so Definition 2 becomes necessary.

**Definition 3 .**

Let *a*_*i*_ ∈ [*I*], *b*_*i*_ ∈ *I*, *i* ∈ *J*, *J* = {1,2, ⋯, *m*}; one defines

(1) $$\begin{array}{c} \overset{}{\underset{i\in J}{⋁}}b_{i}=\sup\left\{b_{i}:i\in J\right\},\;\;\overset{}{\underset{i\in J}{⋀}}b_{i}=\inf\left\{b_{i}:i\in J\right\},\\ \overset{}{\underset{i\in J}{⋁}}a_{i}=\vee \left\{a_{i}:i\in J\right\}=\left[\overset{}{\underset{i\in J}{⋁}}a_{i}^{-},\overset{}{\underset{i\in J}{⋁}}a_{i}^{+}\right],\\ \overset{}{\underset{i\in J}{⋀}}a_{i}=\wedge \left\{a_{i}:i\in J\right\}=\left[\overset{}{\underset{i\in J}{⋀}}a_{i}^{-},\overset{}{\underset{i\in J}{⋀}}a_{i}^{+}\right],\\ \sim a=1-a=\left[1-a^{+},1-a^{-}\right]. \end{array}$$

Obviously, ([*I*], ≤) is a complete lattice, and the triple ([*I*], ∨, ∧) is an algebraic system, which is derived by ([*I*], ≤) with the maximal element [1,1] and the minimum element [0,0].

**Definition 4 .**

Let *U* be a finite and nonempty universe of discourse; then a mapping *A* : *U* → [*I*] is called an interval-valued fuzzy set on *U*. All interval-valued fuzzy sets on *U* are denoted by *F* ^*I*^(*U*). In particular, when *A* = *U*, *A*(*x*) = [1,1], for all *x* ∈ *U*, and when *A* = *∅*, *A*(*x*) = [0,0], for all *x* ∈ *U*.

Similar to fuzzy sets, the operators ⊆, ∩, ∪, and complement of interval-valued fuzzy sets are defined as follows. For all *A*, *B* ∈ *F* ^*I*^(*U*), *A*⊆*B* means *A*(*x*) ≤ *B*(*x*) and for all *x* ∈ *U*, (*A*∩*B*)(*x*) = *A*(*x*)∧*B*(*x*), (*A* ∪ *B*)(*x*) = *A*(*x*)∨*B*(*x*), and (~*A*)(*x*) = 1 − *A*(*x*).

**Definition 5 .**

Let *α* ∈ [*I*], *A* ∈ *F* ^*I*^(*U*). *αA* is called numerical product of *α* and *A* and is defined as (*αA*)(*x*) = *α*∧*A*(*x*), for all *x* ∈ *U*.

**Definition 6 .**

Let *α* ∈ [*I*], *A* ∈ *F* ^*I*^(*U*). *A*_*α*_ = {*x* ∈ *U* : *A*(*x*) ≥ *α*} is called *α*-cut set of *A* and $A_{\bar{\alpha }}=\left\{x\in U:A(x)>\alpha \right\}$ is called strong *α*-cut set of *A*.

**Theorem 7 (the decomposition theorem of the interval-valued fuzzy sets).**

Let *A* ∈ *F* ^*I*^(*U*); then

(2) $$\begin{array}{c} A=\overset{}{\underset{\alpha \in [I]}{⋃}}\alpha A_{\alpha },\;\;A=\overset{}{\underset{\alpha \in [I]}{⋃}}\alpha A_{\bar{\alpha }}. \end{array}$$

**Proof**

For all *x* ∈ *U*,

(3) (⋃α∈[I]αAα)(x)  =⋁α∈[I](α∧Aα(x))  =(⋁α≤A(x)(α∧Aα(x)))∨(⋁α≰A(x)(α∧Aα(x)))  =⋁α≤A(x)(α∧Aα(x))  =⋁α≤A(x)α  =A(x).

Then $A=\underset{}{\cup_{\alpha \in [I]}}\alpha A_{\alpha }$.

Similarly, one can show that $A=\underset{}{\cup_{\alpha \in [I]}}\alpha A_{\bar{\alpha }}$.

**Definition 8 .**

Let *U* and *W* be two finite and nonempty universes of discourse. Then the mapping *IR* : *U* × *W* → [*I*] is called an interval-valued fuzzy relation from *U* to *W*, where *U* × *W* = {(*x*, *y*) : *x* ∈ *U*, *y* ∈ *W*}. When *U* = *W*, *IR* is called an interval-valued fuzzy relation on *U*.

**Remark 9 .**

Obviously, an interval-valued fuzzy relation *IR* from *U* to *W* is an interval-valued fuzzy set denoted by *IR* ∈ *F* ^*I*^(*U* × *W*). So Definitions 4, 5, and 6 and Theorem 7 are still true in the interval-valued fuzzy relation. For example, *IR* ∈ *F* ^*I*^(*U* × *W*) is an interval-valued fuzzy relation. If we see it as an interval-valued fuzzy set, then *IR*_*α*_ = {(*x*, *y*) ∈ *U* × *W* : *R*(*x*, *y*) ≥ *α*}.

**Definition 10 .**

Let *IR* be an interval-valued fuzzy relation from *U* to *W*; then *IR* is said to be serial if and only if for all *x* ∈ *U*, there exists *y* ∈ *W* such that *IR*(*x*, *y*) = [1,1].

**Definition 11 .**

Let *IR* be an interval-valued fuzzy relation on *U*; then *IR* is reflexive if and only if *IR*(*x*, *x*) = [1,1], for all *x* ∈ *U*; *IR* is symmetric if and only if *IR*(*x*, *y*) = *IR*(*y*, *x*), for all *x*, *y* ∈ *U*; *IR* is transitive if and only if $IR(x,z)\geq \underset{}{\vee_{y\in U}}\left(IR\left(x,y\right)\wedge IR\left(y,z\right)\right)$, for all *x*, *z* ∈ *U*; *IR* is Euclidean if and only if $IR\left(y,z\right)\geq \underset{}{\vee_{x\in U}}\left(IR\left(x,y\right)\wedge IR\left(x,z\right)\right)$, for all *y*, *z* ∈ *U*.

One can prove that the binary relation obtained by calculating *α*-cut set or strong *α*-cut set to an interval-valued fuzzy relation, for all *α* ∈ [*I*], still satisfies the corresponding definition of Definition 11 under the classical binary relation; that is, if *IR* is, respectively, reflexive, symmetric, and transitive, then $IR_{\alpha }(IR_{\bar{a}})$ is, respectively, reflexive, symmetric, and transitive under the classical binary relation.

## 3. Generalized Fuzzy Rough Approximation Operators

**Definition 12 .**

Let *U* and *W* be two finite universes of discourse. If *R* is an arbitrary binary fuzzy relation from *U* to *W*, then the triple (*U*, *W*, *R*) is called a generalized fuzzy approximation space.

**Definition 13 .**

Let (*U*, *W*, *R*) be a generalized fuzzy approximation space, for all *x* ∈ *U*; one defines *R*(*x*) = {(*y*, *R*(*x*, *y*)) : *y* ∈ *W*}.

*R*(*x*) is the row of the fuzzy relation which includes *x*, and obviously *R*_*α*_(*x*) = (*R*(*x*))_*α*_.

**Definition 14 .**

Let (*U*, *W*, *R*) be a generalized fuzzy approximation space, for all *α*, *β* ∈ [0,1], *A* ∈ *F*(*W*),

(4) $$\begin{array}{c} {\underline{R}}_{\alpha }\left(A_{\beta }\right)=\left\{x\in U:R_{\alpha }\left(x\right)\subseteq A_{\beta }\right\},\\ {\bar{R}}_{\alpha }\left(A_{\beta }\right)=\left\{x\in U:R_{\alpha }\left(x\right)\cap A_{\beta }\neq \emptyset \right\}. \end{array}$$

${\underline{R}}_{\alpha }(A_{\beta })$ and ${\bar{R}}_{\alpha }(A_{\beta })$ are called (*α*, *β*) lower and upper approximations of *A* with respect to (*U*, *W*, *R*).

**Definition 15 .**

Let (*U*, *W*, *R*) be a generalized fuzzy approximation space, for all *A* ∈ *F*(*W*). One defines

(5) $$\begin{array}{c} \underline{R}\left(A\right)=\overset{}{\underset{\alpha \in I}{⋃}}\alpha {\underline{R}}_{\bar{1-\alpha }}\left(A_{\alpha }\right),\;\;\bar{R}\left(A\right)=\overset{}{\underset{\alpha \in I}{⋃}}\alpha {\bar{R}}_{\alpha }\left(A_{\alpha }\right). \end{array}$$

The pair $\left(\underline{R}\left(A\right),\bar{R}\left(A\right)\right)$ is called the generalized fuzzy rough set of *A* on (*U*, *W*, *R*), and the operators $\underline{R}(A)$ and $\bar{R}(A)$ are called the generalized fuzzy rough lower and upper approximation operators, respectively.

The dual properties are quite useful in proving the properties of the approximation operators. When one intends to prove two dual properties, it suffices to prove one of them, which simplifies the proof procedure. The properties of the lower and upper approximation operators are characterized as follows.

**Theorem 16 .**

Let (*U*, *W*, *R*) be a generalized fuzzy approximation space. Then for all *A* ∈ *F*(*W*), $\underline{R}(A)=\sim \bar{R}(\sim A)$ and $\bar{R}(A)=\sim \underline{R}(\sim A)$.

**Proof**

(1) Note that $\underset{}{\cap_{\alpha \in I}}\left(\underline{\underline{\alpha }}\cup {\underline{R}}_{1-\alpha }\left(A_{\bar{\alpha }}\right)\right)=\underset{}{\cup_{\alpha \in I}}\left(\underline{\underline{\alpha }}\cap {\underline{R}}_{\bar{1-\alpha }}\left(A_{\alpha }\right)\right)$. Here $\underline{\underline{\alpha }}\in F(U)$ and $\underline{\underline{\alpha }}(x)=\alpha$ for all *x* ∈ *U*.

For all *x* ∈ *U*, $x\in {\underline{R}}_{1-\alpha }\left(A_{\bar{\alpha }}\right),\text{that}\;\text{is},\;{\underline{R}}_{1-\alpha }(A_{\bar{\alpha }})(x)=1$, if and only if $R_{1-\alpha }(x)\subseteq A_{\bar{\alpha }}$, such that for all *y* ∈ *W* if *R*(*x*, *y*) ≥ 1 − *α*, then *A*(*y*) > *α*; that is, for all *y* ∈ *W*, *R*(*x*, *y*) < 1 − *α* or *A*(*y*) > *α*.

Hence we have $\underset{}{\wedge_{y\in W}}(\left(1-R\left(x,y\right)\right)\vee A(y))>\alpha$.

For the second case, $x\notin {\underline{R}}_{1-\alpha }(A_{\bar{\alpha }})$; that is, ${\underline{R}}_{1-\alpha }(A_{\bar{\alpha }})(x)=0$ if and only if $\underset{}{\wedge_{y\in W}}\left(\left(1-R\left(x,y\right)\right)\vee A\left(y\right)\right)\leq \alpha$. Since $\underset{}{\wedge_{y\in W}}\left(\left(1-R\left(x,y\right)\right)\vee A\left(y\right)\right)\in I$, there exists *α* ∈ *I*, so that $\alpha =\underset{}{\wedge_{y\in W}}\left(\left(1-R\left(x,y\right)\right)\vee A\left(y\right)\right)$.

Hence,

(6) ⋂α∈I(α__∪R_1−α(Aα¯))(x)  =⋀α∈I(α∨R_1−α(Aα¯)(x))  =⋀y∈W((1−R(x,y))∨A(y)).

Similarly,

(7) ⋃α∈I(α__∩R_1−α¯(Aα))(x)  =⋁α∈I(α∧R_1−α¯(Aα)(x))  =⋀y∈W((1−R(x,y))∨A(y)).

Therefore, $\underset{}{\cap_{\alpha \in I}}(\underline{\underline{\alpha }}\cup {\underline{R}}_{1-\alpha }(A_{\bar{\alpha }}))=\underset{}{\cup_{\alpha \in I}}\left(\underline{\underline{\alpha }}\cap {\underline{R}}_{\bar{1-\alpha }}\left(A_{\alpha }\right)\right)$.

(2) Now, we prove the validity of the relationship $\underline{R}(A)=\sim \bar{R}(\sim A)$. In view of Definition 14, from Theorem  3.2(1) of [18], it follows that

(8) ~R¯(~A)=~⋃α∈I(αR¯α((~A)α))=~⋃α∈I(αR¯α(~A1−α¯))=⋂α∈I(1−α__∪(~R¯α(~A1−α¯)))=⋂α∈I(1−α__∪R_α(A1−α¯))=⋂α∈I(α__∪R_1−α(Aα¯))=⋃α∈I(αR_1−α¯(Aα))=R_(A).

Hence, $\underline{R}(A)=\sim \bar{R}(\sim A)$.

Similarly, $\bar{R}(A)=\sim \underline{R}(\sim A)$.

**Figure 2:**
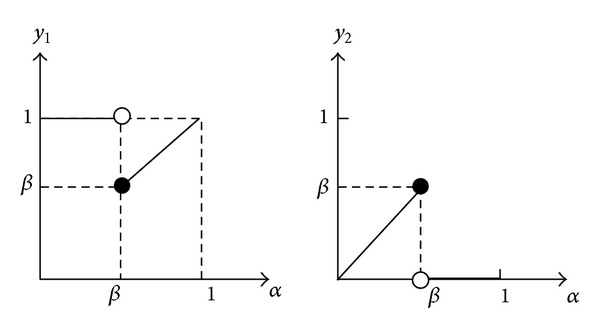
The coordinate frame of functions *y*_1_ and *y*_2_.

Suppose that $y_{1}=\alpha \vee {\underline{R}}_{1-\alpha }(A_{\bar{\alpha }})(x),y_{2}=\alpha \wedge {\underline{R}}_{\bar{1-\alpha }}(A_{\alpha })(x)$ and $\beta =\underset{}{\wedge_{y\in W}}\left(\left(1-R\left(x,y\right)\right)\vee A\left(y\right)\right)$; when the variable *x* is a certain value, the variables *y*_1_ and *y*_2_ are functions of the variable *α*. Refer to Figure 2 for the pertinent detail.

In the proof of Theorem 16, we show that the equation $\underline{R}(A)=\sim \bar{R}(\sim A)$ holds when the minimum of function *y*_1_ is equal to the maximum of function *y*_2_, such that $\underset{}{\cap_{\alpha \in I}}(\underline{\underline{\alpha }}\cup {\underline{R}}_{1-\alpha }(A_{\bar{\alpha }}))=\underset{}{\cup_{\alpha \in I}}(\alpha {\underline{R}}_{\bar{1-\alpha }}(A_{\bar{\alpha }}))$; thus, $\underline{R}(A)=\sim \;\bar{R}(\sim A)$ holds. In [16], the lower approximation operator makes function *y*_2_ equal to zero at the point *β*, which makes the maximum of function *y*_2_ approach *β*, but it does not exist. In this paper, the lower and upper approximation operators in Definition 15 have a better duality.

## 4. Generalized Interval-Valued Fuzzy Rough Sets

**Definition 17 .**

Let *U* and *W* be two finite universes of discourse. If *IR* is an arbitrary binary interval-valued fuzzy relation from *U* to *W*, then the triple (*U*, *W*, *IR*) is called a generalized interval-valued fuzzy approximation space. In particular, when *U* = *W*, the space is denoted by (*U*, *IR*).

**Definition 18 .**

Let (*U*, *W*, *IR*) be a generalized interval-valued fuzzy approximation space, for all *x* ∈ *U*,

(9) $$\begin{array}{c} IR\left(x\right)=\left\{\left(y,IR\left(x,y\right)\right):y\in W\right\}. \end{array}$$

**Definition 19 .**

Let (*U*, *W*, *IR*) be a generalized interval-valued fuzzy approximation space, *A* ∈ *F* ^*I*^(*W*), for all *x* ∈ *U*; one defines

(10) RIF_(A)(x)  =⋀y∈W(~IR(x,y)∨A(y))=[⋀y∈W((1−IR(x,y)+)∨A(y)−),⋀y∈W((1−IR(x,y)−)∨A(y)+)],RIF¯(A)(x)=⋁y∈W(IR(x,y)∧A(y))=[⋁y∈W(IR(x,y)−∧A(y)−),⋁y∈W(IR(x,y)+∧A(y)+)].

The pair $\left(\underline{RIF}(A),\bar{RIF}(A)\right)$ is called the generalized interval-valued fuzzy rough set of *A* with respect to the approximation space (*U*, *W*, *IR*). The operators $\underline{RIF}$ and $\bar{RIF}$ are called the generalized interval-valued fuzzy rough lower and upper approximation operators, respectively.

**Definition 20 .**

Let (*U*, *W*, *IR*) be a generalized interval-valued fuzzy approximation space, for all *α*, *β* ∈ [*I*], *A* ∈ *F* ^*I*^(*W*); one defines

(11) $$\begin{array}{c} {\underline{RIF}}_{\alpha }^{\prime }\left(A_{\beta }\right)=\left\{x\in U:IR_{\alpha }\left(x\right)\subseteq A_{\beta }\right\},\\ {\bar{RIF}}_{\alpha }^{\prime }\left(A_{\beta }\right)=\left\{x\in U:IR_{\alpha }\left(x\right)\cap A_{\beta }\neq \emptyset \right\}. \end{array}$$

${\underline{RIF}}_{\alpha }^{\prime }(A_{\beta })$ and ${\bar{RIF}}_{\alpha }^{\prime }(A_{\beta })$ are called the *α*, *β* lower and upper approximations of *A* with respect to (*U*, *W*, *IR*), respectively.

**Definition 21 .**

Let (*U*, *W*, *IR*) be a generalized interval-valued fuzzy approximation space, *A* ∈ *F* ^*I*^(*W*); one defines

(12) $$\begin{array}{c} {\underline{RIF}}^{\prime }\left(A\right)=\overset{}{\underset{\alpha \in \left[I\right]}{⋃}}\alpha {\underline{RIF}}_{\bar{1-\alpha }}^{\prime }\left(A_{\alpha }\right),\\ {\bar{RIF}}^{\prime }\left(A\right)=\overset{}{\underset{\alpha \in \left[I\right]}{⋃}}\alpha {\bar{RIF}}_{\alpha }^{\prime }\left(A_{\alpha }\right). \end{array}$$

The pair $\left({\underline{RIF}}^{\prime },{\bar{RIF}}^{\prime }\right)$ is called the generalized interval-valued fuzzy rough set of *A* with respect to the approximation space (*U*, *W*, *IR*). The operators ${\underline{RIF}}^{\prime }$ and ${\bar{RIF}}^{\prime }$ are called the generalized interval-valued fuzzy rough lower and upper approximation operators.

**Remark 22 .**

The approximation operators introduced in Definition 20 extend the generalized Dubois fuzzy rough approximation operators from numeric value to intervals. The approximation operators defined in Definition 21 provide the same type of generalization. The approximation operators defined in Definition 21 show the inherent relationship between Pawlak's rough set and interval-valued fuzzy rough sets.

**Lemma 23 .**

Let (*U*, *W*, *IR*) be a generalized interval-valued fuzzy approximation space, *A* ∈ *F* ^*I*^(*W*); then for all $\alpha \in [I],{\bar{RIF}}_{\alpha }^{\prime }(A_{\alpha })\subseteq {\left(\bar{RIF}(A)\right)}_{\alpha }$.

**Proof**

We observe that, for all *x* ∈ *U*, if $x\in {\bar{RIF}}_{\alpha }^{\prime }(A_{\alpha })$, then *IR*_*α*_(*x*)∩*A*_*α*_ ≠ *∅*. This means that there exist *y* ∈ *W*, *IR*(*x*, *y*) ≥ *α*, and *A*(*y*) ≥ *α*.

By the interval-valued operations of Definition 3, we have $\underset{}{\vee_{y\in W}}(IR(x,y)\wedge A(y))\geq \alpha$, so $x\in {\left(\bar{RIF}(A)\right)}_{\alpha }$.

Therefore, ${\bar{RIF}}_{\alpha }^{\prime }(A_{\alpha })\subseteq {\left(\bar{RIF}(A)\right)}_{\alpha }$.

Now, we prove that the reverse of Lemma 23 does not hold. Based on the interval-valued operations, which are defined in Definition 3, there exists *y* ∈ *W*, *IR*(*x*, *y*)∧*A*(*y*) ≥ *α*; that is, there exists *y* ∈ *W*, so that *IR*(*x*, *y*) ≥ *α* and *A*(*y*) ≥ *α* cannot be deduced by $\underset{}{\vee_{y\in W}}\left(IR\left(x,y\right)\wedge A\left(y\right)\right)\geq \alpha$.

Next, we give an example illustrating that the relationship ${\left(\bar{RIF}\left(A\right)\right)}_{\alpha }\subseteq {\bar{RIF}}_{\alpha }^{\prime }(A_{\alpha })$ does not hold.

**Example 24 .**

Suppose that (*U*, *W*, *IR*) is a generalized interval-valued fuzzy approximation space,

(13) $$\begin{array}{c} U=\left\{x_{1},x_{2}\right\},\;\;W=\left\{y_{1},y_{2},y_{3}\right\},\;\;\alpha =\left[0.2,0.6\right],\\ R\left(x_{1}\right)=\frac{\left[0.3,0.5\right]}{y_{1}}+\frac{\left[0.1,0.7\right]}{y_{2}}+\frac{\left[0.1,0.4\right]}{y_{3}},\\ A=\frac{\left[0.2,0.5\right]}{y_{1}}+\frac{\left[0.1,0.9\right]}{y_{2}}+\frac{\left[0.2,0.3\right]}{y_{3}}\text{,} \end{array}$$

since

(14) RIF¯(A)(x1)  =⋁y∈W(R(x1,y)∧A(y))=[0.2,0.7]≥[0.2,0.6], x1∈(RIF¯(A))α.

On the other hand, since *IR*_*α*_(*x*_1_) = *A*_*α*_ = *∅*, we have *IR*_*α*_(*x*_1_)∩*A*_*α*_ = *∅* and we get $x_{1}\notin {\bar{RIF}}_{\alpha }^{\prime }\left(A_{\alpha }\right)$. This shows that $x_{1}\in {\left(\bar{RIF}\left(A\right)\right)}_{\alpha }$, but $x_{1}\notin {\bar{RIF}}_{\alpha }^{\prime }\left(A_{\alpha }\right)$. So ${\left(\bar{RIF}\left(A\right)\right)}_{\alpha }{⊈\bar{RIF}}_{\alpha }^{\prime }\left(A_{\alpha }\right)$.

**Lemma 25 .**

Let (*U*, *W*, *IR*) be a generalized interval-valued fuzzy approximation space, *A* ∈ *F* ^*I*^(*W*); then, for all *α* ∈ [*I*], ${{\underline{RIF}}^{\prime }}_{\bar{1-\alpha }}\left(A_{\alpha }\right)\subseteq {\left(\underline{RIF}\left(A\right)\right)}_{\alpha }$.

**Proof**

Note that, for all *x* ∈ *U*, if $x\in {\underline{RIF}}_{\bar{1-\alpha }}^{\prime }\left(A_{\alpha }\right)$ then $IR_{\bar{1-\alpha }}\left(x\right)\subseteq A_{\alpha }$. This means that, for all *y* ∈ *W*, if *IR*(*x*, *y*) > 1 − *α* then *A*(*y*) ≥ *α*; that is, for all *y* ∈ *W*, *IR*(*x*, *y*) ≤ 1 − *α* or *A*(*y*) ≥ *α*. By the interval-valued operations as in Definition 3, we have $\underset{}{\wedge_{y\in W}}\left(\sim IR\left(x,y\right)\vee A\left(y\right)\right)\geq \alpha$; that is, $\underline{RIF}(A)(x)\geq \alpha$, so $x\in {\left(\underline{RIF}(A)\right)}_{\alpha }$.

Now we prove that the reverse of Lemma 25 is not true. Here we use similar reasoning as already used in Lemma 23. ~*IR*(*x*, *y*)∨*A*(*y*) ≥ *α* means that (1−*IR*(*x*,*y*))^−^∨*A*(*y*)^−^ ≥ *α* ^−^ and (1−*IR*(*x*,*y*))^+^∨*A*(*y*)^+^ ≥ *α* ^+^, which cannot deduce that 1 − *IR*(*x*, *y*) ≥ *α* or *A*(*y*) ≥ *α* in Definition 3. Thus for all *y* ∈ *W*, ~*IR*(*x*, *y*)∨*A*(*y*) ≥ *α*, we cannot deduce that *IR*(*x*, *y*) ≤ 1 − *α* or *A*(*y*) ≥ *α*, and note that, for all *y* ∈ *W*, ~*IR*(*x*, *y*)∨*A*(*y*) ≥ *α* if and only if $\underset{}{\wedge_{y\in W}}\left(\sim IR\left(x,y\right)\vee A\left(y\right)\right)\geq \alpha$. Therefore for all *y* ∈ *W*, *IR*(*x*, *y*) ≤ 1 − *α* or *A*(*y*) ≥ *α* cannot hold.

Next, we show that ${\left(\underline{RIF}\left(A\right)\right)}_{\alpha }\subseteq {\underline{RIF}}_{\bar{1-\alpha }}^{\prime }(A_{\alpha })$ does not hold.

**Example 26 .**

Suppose that (*U*, *W*, *IR*) is a generalized interval-valued fuzzy approximation space,

(15) $$\begin{array}{c} U=\left\{x_{1},x_{2}\right\},\;\;W=\left\{y_{1},y_{2},y_{3}\right\},\;\;\alpha =\left[0.3,0.6\right],\\ IR\left(x_{1}\right)=\frac{\left[0.3,0.8\right]}{y_{1}}+\frac{\left[0.4,0.9\right]}{y_{2}}+\frac{\left[0.5,0.6\right]}{y_{3}},\\ A=\frac{\left[0.4,0.5\right]}{y_{1}}+\frac{\left[0.3,0.4\right]}{y_{2}}+\frac{\left[0.4,0.9\right]}{y_{3}}, \end{array}$$

since

(16) RIF_(A)(x1)=⋀y∈W(~IR(x1,y)∨A(y))=[0.3,0.6]≥[0.3,0.6], x1∈(RIF_(A))α.

On the other hand, since $IR_{\bar{1-\alpha }}\left(x_{1}\right)=y_{2},A_{\alpha }=y_{3}$, we have $IR_{\bar{1-\alpha }}(x_{1})⊈A_{\alpha }$; hence $x_{1}\notin {\underline{RIF}}_{\bar{1-\alpha }}^{\prime }(A_{\alpha })$. This shows that $x_{1}\in {\left(\underline{RIF}\left(A\right)\right)}_{\alpha }$, but $x_{1}\notin {\underline{RIF}}_{\bar{1-\alpha }}^{\prime }(A_{\alpha })$. Therefore ${\left(\underline{RIF}\left(A\right)\right)}_{\alpha }⊈{\underline{RIF}}_{\bar{1-\alpha }}^{\prime }(A_{\alpha })$.

**Theorem 27 .**

Let (*U*, *W*, *IR*) be a generalized interval-valued fuzzy approximation space, *A* ∈ *F* ^*I*^(*W*); then $\bar{RIF}(A)={\bar{RIF}}^{\prime }(A)$.

**Proof**

According to Theorem 7, we have $\bar{RIF}(A)=\underset{}{\cup_{\alpha \in [I]}}\alpha {\left(\bar{RIF}(A)\right)}_{\alpha }$, and from Lemma 23, we see that ${\bar{RIF}}_{\alpha }^{\prime }(A_{\alpha })\subseteq {\left(\bar{RIF}(\text{A})\right)}_{\alpha }$, for all *α* ∈ [*I*].

Therefore, ${\bar{RIF}}^{\prime }(A)\subseteq \bar{RIF}(A)$.

Next we prove that $\bar{RIF}(A)\subseteq {\bar{RIF}}^{\prime }(A)$.

In fact, for all *x* ∈ *U*, *y* ∈ *W*, there exists *α* = *IR*(*x*, *y*)∧*A*(*y*)∈[*I*], such that *y* ∈ *IR*_*α*_(*x*)∩*A*_*α*_. We observe that *y* ∈ *IR*_*α*_(*x*)∩*A*_*α*_ means that *IR*_*α*_(*x*)∩*A*_*α*_ ≠ *∅*, which can deduce that $x\in {\bar{RIF}}_{\alpha }^{\prime }(A_{\alpha })$; that is, ${\bar{RIF}}_{\alpha }^{\prime }(A_{\alpha })(x)=1$; hence $\alpha \wedge {\bar{RIF}}_{\alpha }^{\prime }(A_{\alpha })(x)=\alpha =IR(x,y)\wedge A(y)$.

So, for arbitrary value of *y*, ${\bar{RIF}}^{\prime }(A)(x)=\underset{}{\vee_{\alpha \in [I]}}(\alpha \wedge {\bar{RIF}}_{\alpha }^{\prime }(A_{\alpha })(x))\geq \underset{}{\vee_{y\in W}}(IR(x,y)\wedge A(y))=\bar{RIF}(A)(x)$, which yields $\bar{RIF}(A)\subseteq {\bar{RIF}}^{\prime }(A)$.

Therefore, $\bar{RIF}(A)={\bar{RIF}}^{\prime }(A)$.

**Theorem 28 .**

Let (*U*, *W*, *IR*) be a generalized interval-valued fuzzy approximation space, *A* ∈ *F* ^*I*^(*W*); then $\underline{RIF}(A)={\underline{RIF}}^{\prime }(A)$.

**Proof**

In view of Theorem 7, we have $\underline{RIF}(A)=\underset{}{\cup_{\alpha \in [I]}}\alpha {\left(\underline{RIF}(A)\right)}_{\alpha }$, and from Lemma 25, ${\underline{RIF}}_{\bar{1-\alpha }}^{\prime }(A_{\alpha })\subseteq {\left(\underline{RIF}(A)\right)}_{\alpha }$, for all *α* ∈ [*I*].

Then ${\underline{RIF}}^{\prime }(A)\subseteq \underline{RIF}(A)$.

Now we prove that $\underline{RIF}\left(A\right)\subseteq {\underline{RIF}}^{\prime }\left(A\right)$. For all *x* ∈ *U*, suppose that

(17) α1=[0,⋀y∈W(~IR(x,y)∨A(y))+],α2=[⋀y∈W(~IR(x,y)∨A(y))−,⋀y∈W(~IR(x,y)∨A(y))−].

(1) We verify that $x\in {\underline{RIF}}_{\bar{1-\alpha_{1}}}^{\prime }\left(A_{\alpha_{1}}\right)$. Let

(18) $$\begin{array}{c} \alpha_{1}=\left[0,\overset{}{\underset{y\in W}{⋀}}\left(\left(1-IR{\left(x,y\right)}^{-}\right)\vee A{\left(y\right)}^{+}\right)\right],\\ 1-\alpha_{1}=\left[\overset{}{\underset{y\in W}{⋁}}\left(IR{\left(x,y\right)}^{-}\wedge \left(1-A{\left(y\right)}^{+}\right)\right),1\right]. \end{array}$$

Note that, for all *y*_0_ ∈ *W*, $y_{0}\in IR_{\bar{1-\alpha_{1}}}(x)$, such that *IR*(*x*, *y*_0_) > 1 − *α*_1_, and from (1−*α*_1_)^+^ = 1, we have *IR*(*x*,*y*_0_)^−^ > ∨_*y*∈*W*_(*IR*(*x*,*y*)^−^∧(1 − *A*(*y*)^+^)).

Further from *y*_0_ ∈ *W*, we have *IR*(*x*,*y*_0_)^−^ > *IR*(*x*,*y*_0_)^−^∧(1 − *A*(*y*_0_)^+^)  .

Therefore we obtain that *IR*(*x*,*y*_0_)^−^ > 1 − *A*(*y*_0_)^+^  and  1 − *A*(*y*_0_)^+^ = *IR*(*x*,*y*_0_)^−^∧(1 − *A*(*y*_0_)^+^).

Because $IR{\left(x,y_{0}\right)}^{-}\wedge \left(1-A{\left(y_{0}\right)}^{+}\right)\leq \underset{}{\vee_{y\in W}}\left(IR{\left(x,y\right)}^{-}\wedge \left(1-A{\left(y\right)}^{+}\right)\right)$, we have $1-A{\left(y_{0}\right)}^{+}\leq \underset{}{\vee_{y\in W}}\left(IR{\left(x,y\right)}^{-}\wedge \left(1-A{\left(y\right)}^{+}\right)\right)$, such that $A{\left(y_{0}\right)}^{+}\geq \underset{}{\wedge_{y\in W}}\left(\left(1-IR{\left(x,y\right)}^{-}\right)\vee A{\left(y\right)}^{+}\right)$, and from *α*_1_^−^ = 0, we get *A*(*y*_0_) ≥ *α*_1_; that is, *y*_0_ ∈ *A*_*α*_1__.

So, for arbitrary value of *y*_0_, $IR_{\bar{1-\alpha_{1}}}(x)\subseteq A_{\alpha_{1}}$; that is, $x\in {\underline{RIF}}_{\bar{1-\alpha_{1}}}^{\prime }(A_{\alpha_{1}})$.

(2) Similar to the proof shown in (1), we have $x\in {\underline{RIF}}_{\bar{1-\alpha_{2}}}^{\prime }(A_{\alpha_{2}})$. Note that

(19) RIF_′(A)(x) =⋁α∈[I](α∧RIF_1−α¯′(Aα)(x)) ≥(α1∧RIF_1−α1¯′(Aα1)(x))∨(α2∧RIF_1−α2¯′(Aα2)(x)) =α1∨α2 =[⋀y∈W(~IR(x,y)∨A(y))−,⋀y∈W(~IR(x,y)∨A(y))+] =RIF_(A)(x).

For any *x*, $\underline{RIF}(A)\subseteq {\underline{RIF}}^{\prime }(A)$.

Therefore, $\underline{RIF}(A)={\underline{RIF}}^{\prime }(A)$.

**Remark 29 .**

According to Theorems 27 and 28, ${\underline{RIF}}^{\prime }$ and ${\bar{RIF}}^{\prime }$ satisfy the property of duality.

**Theorem 30 .**

Let (*U*, *W*, *IR*) be a generalized interval-valued fuzzy approximation space, *A* ∈ *F* ^*I*^(*W*); then $\sim \underline{RIF}(A)=\bar{RIF}(\sim A),\sim \bar{RIF}(A)=\underline{RIF}(\sim A)$.

**Proof**

We observe that, for all *x* ∈ *U*,

(20) RIF¯(~A)(x)  =[⋁y∈W(IR(x,y)∧(~A)(y))−,   ⋁y∈W(IR(x,y)∧(~A)(y))+]  =[⋁y∈W(IR(x,y)−∧(1−A(y)+)),   ⋁y∈W(IR(x,y)+∧(1−A(y)−))]  =1−[⋀y∈W((1−IR(x,y)+)∨A(y)−),     ⋀y∈W((1−IR(x,y)−)∨A(y)+)]  =1−[⋀y∈W((1−IR(x,y))−∨A(y)−),     ⋀y∈W((1−IR(x,y))+∨A(y)+)]  =1−[⋀y∈W((1−IR(x,y))∨A(y))−,     ⋀y∈W((1−IR(x,y))∨A(y))+]  =~RIF_(A)(x).

Hence, $\sim \underline{RIF}(A)=\bar{RIF}(\sim A)$.

Similarly, $\sim \bar{RIF}(A)=\underline{RIF}(\sim A)$.

## 5. Properties of the Approximation Operators

**Theorem 31 .**

Let (*U*, *W*, *IR*) be a generalized interval-valued fuzzy approximation space; then the lower approximation operator ${\underline{RIF}}^{\prime }$ and the upper approximation operator ${\bar{RIF}}^{\prime }$ satisfy the following properties.

For all *A*, *B* ∈ *F* ^*I*^(*W*), *a* ∈ [*I*],

1. ${\underline{RIF}}^{\prime }(A\cup \underline{\underline{a}})={\underline{RIF}}^{\prime }(A)\cup \underline{\underline{a}}$,
2. ${\bar{RIF}}^{\prime }\left(A\cap \underline{\underline{a}}\right)={\bar{RIF}}^{\prime }\left(A\right)\cap \underline{\underline{a}}$;
3. ${\bar{RIF}}^{\prime }(A\cup B)={\bar{RIF}}^{\prime }(A)\cup {\bar{RIF}}^{\prime }(B)$,
4. ${\underline{RIF}}^{\prime }(A\cap B)={\underline{RIF}}^{\prime }(A)\cap {\underline{RIF}}^{\prime }(B)$;
5. $if\;A\subseteq B\;then\;{\underline{RIF}}^{\prime }(A)\subseteq {\underline{RIF}}^{\prime }(B)$,
6. $and\;{\bar{RIF}}^{\prime }(A)\subseteq {\bar{RIF}}^{\prime }(B)$;
7. ${\underline{RIF}}^{\prime }(A\cup B)⊇{\underline{RIF}}^{\prime }(A)\cup {\underline{RIF}}^{\prime }(B)$,
8. ${\bar{RIF}}^{\prime }(A\cap B)\subseteq {\bar{RIF}}^{\prime }(A)\cap {\bar{RIF}}^{\prime }(B)$.

Here $\underline{\underline{a}}$ is a constant interval-valued fuzzy set; that is, $\underline{\underline{a}}(x)=a$, for all *x* ∈ *U* and *x* ∈ *W*.

**Proof**

(1) We prove that ${\underline{RIF}}^{\prime }(A\cup \underline{\underline{a}})={\underline{RIF}}^{\prime }(A)\cup \underline{\underline{a}}$.

For all *x* ∈ *U*, let

(21) $$\begin{array}{c} D_{1}=\left\{\alpha \in \left[I\right]:\forall y\in IR_{\bar{1-\alpha }}\left(x\right),A\left(y\right)\geq \alpha \;\text{or}\;a\geq \alpha \right\},\\ D_{2}=\left\{\alpha \in \left[I\right]:\forall y\in IR_{\bar{1-\alpha }}\left(x\right),A\left(y\right)\vee a\geq \alpha \right\}, \end{array}$$

where ∀*x* is “for all *x*” and ∃*x* is “there exists *x*,” which are the same as follows.

Obviously, $D_{1}\subseteq D_{2},\underset{}{\vee_{\alpha \in D_{1}}}\alpha \leq \underset{}{\vee_{\alpha \in D_{2}}}\alpha$. Set *D*_3_ = *D*_2_ − *D*_1_, for all *β* ∈ *D*_3_; two cases appear:

(22) $$\begin{array}{c} \beta^{+}\leq a^{+},\;\;\beta^{-}\leq \overset{}{\underset{y\in R_{\bar{1-\alpha }}\left(x\right)}{⋀}}A{\left(y\right)}^{-} \end{array}$$

or

(23) $$\begin{array}{c} \beta^{-}\leq a^{-},\;\;\beta^{+}\leq \overset{}{\underset{y\in R_{\bar{1-\alpha }}\left(x\right)}{⋀}}A{\left(y\right)}^{+}. \end{array}$$

For the first case, suppose $b=\left[\underset{}{\wedge_{y\in R_{\bar{1-\alpha }}(x)}}A{\left(y\right)}^{-},\underset{}{\wedge_{y\in R_{\bar{1-\alpha }}(x)}}A{\left(y\right)}^{+}\right]$, because *a*, *b* ∈ *D*_1_; we have $\beta \leq a\vee b\leq \underset{}{\vee_{\alpha \in D_{1}}}\alpha$. The proof for the second case is similar.

For arbitrary *β*, it is easy to see that $\underset{}{\vee_{\alpha \in D_{3}}}\alpha \leq \underset{}{\vee_{\alpha \in D_{1}}}\alpha$, so $\underset{}{\vee_{\alpha \in D_{2}}}\alpha =\underset{}{\vee_{\alpha \in D_{1}}}\alpha$:

(24) RIF_′(A∪a__)(x)  =⋁α∈[I](α∧RIF_1−α¯′(A∪a__)α(x))  =∨{α∈[I]:IR1−α¯(x)⊆(A∪a__)α}  =∨{α∈[I]:∀y∈IR1−α¯(x),A(y)∨a≥α}  =∨{α∈[I]:∀y∈IR1−α¯(x),A(y)≥α  or  a≥α}  =a∨(∨{α∈[I]:∀y∈IR1−α¯(x),A(y)≥α})  =a∨RIF_′(A)(x)  =(a__∪RIF_′(A))(x).

Hence, ${\underline{RIF}}^{\prime }(A\cup \underline{\underline{a}})={\underline{RIF}}^{\prime }(A)\cup \underline{\underline{a}}$.

Similarly, ${\bar{RIF}}^{\prime }(A\cap \underline{\underline{a}})={\bar{RIF}}^{\prime }(A)\cap \underline{\underline{a}}$.

(2) We verify ${\bar{RIF}}^{\prime }(A\cup B)={\bar{RIF}}^{\prime }(A)\cup {\bar{RIF}}^{\prime }(B)$.

For all *x* ∈ *U*, let

(25) $$\begin{array}{c} D_{4}=\left\{\alpha \in \left[I\right]:\exists y\in IR_{\alpha }\left(x\right),A\left(y\right)\geq \alpha \;\text{or}\;B\left(y\right)\geq \alpha \right\};\\ D_{5}=\left\{\alpha \in \left[I\right]:\exists y\in IR_{\alpha }\left(x\right),A\left(y\right)\vee B\left(y\right)\geq \alpha \right\}, \end{array}$$

$\underset{}{\vee_{\alpha \in D_{5}}}\alpha =\underset{}{\vee_{\alpha \in D_{4}}}\alpha$ holds by using similar arguments as in (1).

We observe that

(26) RIF¯′(A∪B)(x) =⋁α∈[I](α∧RIF¯α′(A∪B)α(x)) =∨{α∈[I]:IRα(x)∩(A∪B)α≠∅} =∨{α∈[I]:∃y∈IRα(x),(A∪B)(y)≥α} =∨{α∈[I]:∃y∈IRα(x),A(y)∨B(y)≥α} =∨{α∈[I]:∃y∈IRα(x),A(y)≥α  or  B(y)≥α} =(∨{α∈[I]:∃y∈IRα(x),A(y)≥α})  ∨(∨{α∈[I]:∃y∈IRα(x),B(y)≥α}) =(⋁α∈[I](α∧RIF¯α′(A)α(x)))  ∨(⋁α∈[I](α∧RIF¯α′(B)α(x))) =RIF¯′(A)(x)∨RIF¯′(B)(x) =(RIF¯′(A)∪RIF¯′(B))(x),

which yields that ${\underline{RIF}}^{\prime }(A\cup B)={\underline{RIF}}^{\prime }(A)\cup {\underline{RIF}}^{\prime }(B)$.

Similarly, ${\underline{RIF}}^{\prime }(A\cap B)={\underline{RIF}}^{\prime }(A)\cap {\underline{RIF}}^{\prime }(B)$.

(3) We prove that if *A*⊆*B* then ${\underline{RIF}}^{\prime }(A)\subseteq {\underline{RIF}}^{\prime }(B)$. If *A*⊆*B*, then *A*_*α*_⊆*B*_*α*_. According to Definition 20 and Theorem  3.2(4) of [18], we have ${\underline{RIF}}_{\bar{1-\alpha }}^{\prime }(A_{\alpha })\subseteq {\underline{RIF}}_{\bar{1-\alpha }}^{\prime }(B_{\alpha })$, so $\underset{}{\cup_{\alpha \in [I]}}\alpha {\underline{RIF}}_{\bar{1-\alpha }}^{\prime }(A_{\alpha })\subseteq \underset{}{\cup_{\alpha \in [I]}}\alpha {\underline{RIF}}_{\bar{1-\alpha }}^{\prime }(B_{\alpha })$; that is, ${\underline{RIF}}^{\prime }(A)\subseteq {\underline{RIF}}^{\prime }(B)$.

Similarly, if *A*⊆*B*, then ${\bar{RIF}}^{\prime }(A)\subseteq {\bar{RIF}}^{\prime }(B)$.

(4) From (3), one immediately obtains (4).

**Remark 32 .**

From Theorem 31 (1), one can see that ${\underline{RIF}}^{\prime }(W)=U,\;{\bar{RIF}}^{\prime }(\emptyset )=\emptyset$.

**Theorem 33 .**

Let (*U*, *W*, *IR*) be a generalized interval-valued fuzzy approximation space; then the following conditions are equivalent:

1. *IR* is serial;
2. ${\underline{RIF}}^{\prime }(A)\subseteq {\bar{RIF}}^{\prime }(A)$, for all *A*⊆*F* ^*I*^(*W*);
3. ${\bar{RIF}}^{\prime }(W)=U$;
4. ${\underline{RIF}}^{\prime }(\emptyset )=\emptyset$.

**Theorem 34 .**

Let (*U*, *IR*) be a generalized interval-valued fuzzy approximation space; then the following conditions are equivalent:

1. *IR* is reflexive;
2. ${\underline{RIF}}^{\prime }(A)\subseteq A$, for all *A*⊆*F* ^*I*^(*W*);
3. $A\subseteq {\bar{RIF}}^{\prime }(A)$, for all *A*⊆*F* ^*I*^(*W*).

**Lemma 35 .**

Let (*U*, *W*, *IR*) be a generalized interval-valued fuzzy approximation space; then the following properties hold:

1. ${\bar{RIF}}^{\prime }(1_{\left\{y\right\}})(x)=IR(x,y)$, (*x*, *y*) ∈ *U* × *W*;
2. ${\underline{RIF}}^{\prime }(1_{W\setminus \{y\}})(x)=1-IR(x,y)$,(*x*, *y*) ∈ *U* × *W*.

Here 1_*A*_ is an interval-valued fuzzy set which gets interval value [1,1] in the set *A* and interval value [0,0] in the set ~*A*, respectively.

**Remark 36 .**

The proofs of Theorems 33 and 34 as well as Lemma 35 are similar to Theorems 3.8, 3.9, and  3.7 in [16], respectively; it suffices to change point values to interval values in the proof.

**Lemma 37 .**

Let (*U*, *IR*) be a generalized interval-valued fuzzy approximation space and *A* is an interval-valued fuzzy set on *U*; then ∀*α* ∈ [*I*], ${\bar{RIF}}_{\alpha }^{\prime }(A_{\alpha })\subseteq {\left({\bar{RIF}}^{\prime }\left(A\right)\right)}_{\alpha }$, and ${\underline{RIF}}_{\bar{1-\alpha }}^{\prime }(A_{\alpha })\subseteq {\left({\underline{RIF}}^{\prime }\left(A\right)\right)}_{\alpha }$.

**Proof**

Clearly,

(27) RIF¯α′(Aα)=(αRIF¯α′(Aα))α⊆(αRIF¯α′(Aα)∪(⋃β∈[I]−αβRIF¯β′(Aβ)))α=(⋃β∈[I]βRIF¯β′(Aβ))α=(RIF¯′(A))α.

Hence, ${\bar{RIF}}_{\alpha }^{\prime }\left(A_{\alpha }\right)\subseteq {\left({\bar{RIF}}^{\prime }(A)\right)}_{\alpha }$.

Similarly, ${\underline{RIF}}_{\bar{1-\alpha }}^{\prime }\left(A_{\alpha }\right)\subseteq {\left({\underline{RIF}}^{\prime }\left(A\right)\right)}_{\alpha }$.

**Remark 38 .**

${\bar{RIF}}_{\alpha }^{\prime }\left(A_{\alpha }\right)={\left({\bar{RIF}}^{\prime }\left(A\right)\right)}_{\alpha }$ and ${\underline{RIF}}_{\bar{1-\alpha }}^{\prime }\left(A_{\alpha }\right)={\left({\underline{RIF}}^{\prime }\left(A\right)\right)}_{\alpha }$ hold for the fuzzy rough set in Lemma 37, but these are not true for the interval-valued fuzzy rough set. The reason is that the two interval values cannot always be comparable. Next, we give two examples to visualize this effect.

**Example 39 .**

Suppose that (*U*, *IR*) is a generalized interval-valued fuzzy approximation space, where *U* = {*x*_1_, *x*_2_}, *IR*(*x*_1_) = [0.5,0.5]/*x*_1_ + [0.3,0.7]/*x*_2_, *A* = [0.9,1]/*x*_1_ + [0.7,0.9]/*x*_2_,   *α* = [0.4,0.6], *β*_1_ = [0.5,0.5], and *β*_2_ = [0.3,0.7]. Since *IR*_*α*_(*x*_1_) = *∅*, *A*_*α*_ = {*x*_1_, *x*_2_}, we have *IR*_*α*_(*x*_1_)∩*A*_*α*_ = *∅*. Hence $x_{1}\notin {\bar{RIF}}_{\alpha }^{\prime }\left(A_{\alpha }\right)$.

On the other hand, since *IR*_*β*_1__(*x*_1_) = {*x*_1_}, *A*_*β*_1__ = {*x*_1_, *x*_2_}, *IR*_*β*_2__(*x*_1_) = {*x*_2_}, *A*_*β*_2__ = {*x*_1_, *x*_2_}, we see that $x_{1}\in {\bar{RIF}}_{\beta_{1}}^{\prime }(A_{\beta_{1}})$ and $x_{1}\in {\bar{RIF}}_{\beta_{2}}^{\prime }(A_{\beta_{2}})$get $\beta_{1}\wedge {\bar{RIF}}_{\beta_{1}}^{\prime }(A_{\beta_{1}})(x_{1})=\beta_{1}$ and $\beta_{2}\wedge {\bar{RIF}}_{\beta_{2}}^{\prime }(A_{\beta_{2}})(x_{1})=\beta_{2}$.

Note that $\underset{}{\vee_{\beta \in [I]}}\left(\beta \wedge {\bar{RIF}}_{\beta }^{\prime }\left(A_{\beta }\right)\left(x_{1}\right)\right)\geq \beta_{1}\vee \beta_{2}>\alpha$; then ${\bar{RIF}}^{\prime }\left(A\right)\left(x_{1}\right)>\alpha$. Hence, $x_{1}\in {\left({\bar{RIF}}^{\prime }(A)\right)}_{\alpha }$.

**Example 40 .**

Suppose that (*U*, *IR*) is a generalized interval-valued fuzzy approximation space, where *U* = {*x*_1_, *x*_2_, *x*_3_}, *IR*(*x*_1_) = [0.3,0.7]/*x*_1_ + [0.1,0.8]/*x*_2_ + [0.5,0.5]/*x*_3_, *A* = [0.5,0.7]/*x*_1_ + [0.6,1]/*x*_2_ + [0.7,1]/*x*_3_,*α* = [0.4,0.8], *β*_1_ = [0.2,1], *β*_2_ = [0.5,0.6].

Since $IR_{\bar{1-\alpha }}\left(x_{1}\right)=\{x_{1}\},A_{\alpha }=\{x_{2},x_{3}\}$, we have $IR_{\bar{1-\alpha }}(x_{1})⊈A_{\alpha }$; hence, $x_{1}\notin {\underline{RIF}}_{\bar{1-\alpha }}^{\prime }(A_{\alpha })$.

On the other hand, since $IR_{\bar{1-\beta_{1}}}(x_{1})=\{x_{2}\},A_{\beta_{1}}=\{x_{2},x_{3}\},IR_{\bar{1-\beta_{2}}}(x_{1})=\{x_{3}\},A_{\beta_{2}}=\{x_{2},x_{3}\}$, we conclude that $x_{1}\in {\underline{RIF}}_{\bar{1-\beta_{1}}}^{\prime }(A_{\beta_{1}})\;\text{and}\;x_{1}\in {\underline{RIF}}_{\bar{1-\beta_{2}}}^{\prime }\;(A_{\beta_{2}})$ get $\beta_{1}\wedge {\underline{RIF}}_{\bar{1-\beta_{1}}}^{\prime }(A_{\beta_{1}})(x_{1})=\beta_{1}$ and $\beta_{2}\wedge {\underline{RIF}}_{\bar{1-\beta_{2}}}^{\prime }(A_{\beta_{2}})(x_{1})=\beta_{2}$.

Note that $\underset{}{\vee_{\beta \in [I]}}\left(\beta \wedge {\underline{RIF}}_{\bar{1-\beta }}^{\prime }\left(A_{\beta }\right)\left(x_{1}\right)\right)\geq \beta_{1}\vee \beta_{2}>\alpha$; then ${\underline{RIF}}^{\prime }(A)(x_{1})>\alpha$.

Therefore, $x_{1}\in {\left({\underline{RIF}}^{\prime }(A)\right)}_{\alpha }$.

**Theorem 41 .**

Let (*U*, *IR*) be a generalized interval-valued fuzzy approximation space; then the following conditions are equivalent:

1. *IR* is transitive;
2. ${\underline{RIF}}^{\prime }(A)\subseteq {\underline{RIF}}^{\prime }\left({\underline{RIF}}^{\prime }\left(A\right)\right)$, for all *A* ∈ *F* ^*I*^(*U*);
3. ${\bar{RIF}}^{\prime }(A)\subseteq {\bar{RIF}}^{\prime }\left({\bar{RIF}}^{\prime }\left(A\right)\right)$, for all *A* ∈ *F* ^*I*^(*U*).

**Proof**

(1)⇒(2) For all *A* ∈ *F* ^*I*^(*U*), from Definition 20, Lemma 37, and Theorem 3.6 of [18], we have

(28) RIF_′(RIF_′(A))=⋃α∈[I]αRIF_1−α¯′((RIF_′(A))α)⊇⋃α∈[I]αRIF_1−α¯′((RIF_1−α¯′(Aα)))⊇⋃α∈[I]αRIF_1−α¯′(Aα)=RIF_′(A).

Hence ${\underline{RIF}}^{\prime }(A)\subseteq {\underline{RIF}}^{\prime }\left({\underline{RIF}}^{\prime }\left(A\right)\right)$.

(3)⇒(1) For all *x*, *y*, *z* ∈ *U*, let

(29) D6={α∈[I]:∃u∈U,IR(x,u)≥α,RIF¯′(1{z})(u)=IR(u,z)≥α},D7(y)=IR(x,y)∧IR(y,z),   ∀y∈U.

For all *y* ∈ *U*, suppose that *α* = *D*_7_(*y*) = *IR*(*x*, *y*)∧*IR*(*y*, *z*); then *IR*(*x*, *y*) ≥ *α*, *IR*(*y*, *z*) ≥ *α*; hence *α* ∈ *D*_6_ and by the arbitrary *y*, $\underset{}{\vee_{y\subseteq U}}D_{7}(y)\leq \underset{}{\vee_{\alpha \in D_{6}}}\alpha$.

For all *α* ∈ *D*_6_, there exists *y* ∈ *U*, such that *α* ≤ *IR*(*x*, *y*)∧*IR*(*y*, *z*). For arbitrary *α*, $\underset{}{\vee_{y\subseteq U}}D_{7}(y)\geq \underset{}{\vee_{\alpha \in D_{6}}}\alpha$, so $\underset{}{\vee_{y\subseteq U}}D_{7}(y)=\underset{}{\vee_{\alpha \in D_{6}}}\alpha$. We observe that

(30) RIF¯′(RIF¯′(1{z}))(x)  =∨{α∈[I]:x∈RIF¯α′(RIF¯′(1{z}))α}  =∨{α∈[I]:IRα(x)∩(RIF¯′(1{z}))α≠∅}  =∨{α∈[I]:∃u∈U,IR(x,u)≥α,(RIF¯′(1{z}))(u)=IR(u,z)≥α}  =⋁y∈U(IR(x,y)∧IR(y,z)).

Hence, by Lemma 35 (1), we have ${\bar{RIF}}^{\prime }\left({\bar{RIF}}^{\prime }\left(1_{\left\{z\right\}}\right)\right)(x)\leq {\bar{RIF}}^{\prime }\left(1_{\left\{z\right\}}\right)\left(x\right)=IR(x,z)$; then $IR\left(x,z\right)\geq \underset{}{\vee_{y\in U}}\left(IR\left(x,y\right)\wedge IR\left(y,z\right)\right)$. Therefore, *IR* is transitive.

(2)⇔(3) This conclusion follows immediately from the duality.

**Remark 42 .**

In [18], if *IR* is symmetric, then the approximation operators satisfy $A\subseteq \underline{R}\bar{R}(A)$ and $\bar{R}\underline{R}(A)\subseteq A$ for all *A*⊆*U*; if *IR* is Euclidean, then the approximation operators satisfy $\bar{R}(A)\subseteq \underline{R}\bar{R}(A)$ and $\bar{R}\underline{R}(A)\subseteq \underline{R}(\text{A})$ for all *A*⊆*U*. These properties do not hold in the interval-valued fuzzy rough sets. Next, we give a counterexample to show it.

**Example 43 .**

Suppose that (*U*, *IR*) is a generalized interval-valued fuzzy approximation space, *U* = {*x*_1_, *x*_2_, *x*_3_}, and

(31) $$\begin{array}{c} IR=\left(\begin{array}{ccc} \left[0.9,1\right] & \left[0.6,0.6\right] & \left[0.1,0.6\right]\\ \left[0.6,0.6\right] & \left[0.8,0.9\right] & \left[0.1,0.9\right]\\ \left[0.1,0.6\right] & \left[0.1,0.9\right] & \left[0.1,1\right] \end{array}\right),\\ A=\frac{\left[0.3,0.4\right]}{x_{1}}+\frac{\left[0.4,0.6\right]}{x_{2}}+\frac{\left[0.2,0.8\right]}{x_{3}},\\ {\bar{RIF}}^{\prime }\left(A\right)=\frac{\left[0.4,0.6\right]}{x_{1}}+\frac{\left[0.4,0.8\right]}{x_{2}}+\frac{\left[0.1,0.8\right]}{x_{3}},\\ {\underline{RIF}}^{\prime }\left({\bar{RIF}}^{\prime }\left(A\right)\right)=\frac{\left[0.4,0.6\right]}{x_{1}}+\frac{\left[0.1,0.6\right]}{x_{2}}+\frac{\left[0.1,0.9\right]}{x_{3}}. \end{array}$$

From Definition 11, *IR* is symmetric and Euclidean, but ${\bar{RIF}}^{\prime }(A)\subseteq {\underline{RIF}}^{\prime }({\bar{RIF}}^{\prime }(A))$ and $A\subseteq {\underline{RIF}}^{\prime }({\bar{RIF}}^{\prime }(A))$ do not hold. According to the duality, ${\bar{RIF}}^{\prime }({\underline{RIF}}^{\prime }(A))\subseteq {\underline{RIF}}^{\prime }(A)$ and ${\bar{RIF}}^{\prime }({\underline{RIF}}^{\prime }(A))\subseteq A$ are not true.

**Theorem 44 .**

Let (*U*, *IR*) be a generalized interval-valued fuzzy approximation space; then the following conditions are equivalent:

1. $IR(x,z)\leq \underset{}{\wedge_{y\in U}}\left(\left(1-IR\left(x,y\right)\right)\vee IR\left(y,z\right)\right),x,z\in U$;
2. ${\bar{RIF}}^{\prime }({\underline{RIF}}^{\prime }(A))\subseteq {\underline{RIF}}^{\prime }(A),A\in F^{I}(U)$;
3. ${\bar{RIF}}^{\prime }(A)\subseteq {\underline{RIF}}^{\prime }({\bar{RIF}}^{\prime }(A)),A\in F^{I}(U)$.

**Proof**

(2)⇒(1) For all *x*, *z* ∈ *U*, we have

(32) RIF¯′(RIF_′(1U∖{z}))(x)=∨{α∈[I]:x∈RIF¯α′(RIF_′(1U∖{z}))α}=∨{α∈[I]:IRα(x)∩(RIF_′(1U∖{z}))α≠∅}=∨{α∈[I]:∃y∈U,IR(x,y)≥α,RIF_′(1U∖{z})(y)≥α}=∨{α∈[I]:∃y∈U,IR(x,y)≥α,1−IR(y,z)≥α}=∨{α∈[I]:∃y∈U,IR(x,y)∧(1−IR(y,z))≥α}=⋁y∈U(IR(x,y)∧(1−IR(y,z))).

By Lemma 35 (2), we get ${\underline{RIF}}^{\prime }(1_{U\setminus \{z\}})(x)=1-IR(x,z)\;$.

Hence, $\underset{}{\vee_{y\in U}}\left(IR\left(x,y\right)\wedge \left(1-IR\left(y,z\right)\right)\right)\leq 1-IR(x,z)$.

At the same time, we have

(33) IR(x,z)≤1−⋁y∈U(IR(x,y)∧(1−IR(y,z)))=[1,1]−[⋁y∈U(IR(x,y)−∧(1−IR(y,z)+)),⋁y∈U(IR(x,y)+∧(1−IR(y,z)−))]=[1−⋁y∈U(IR(x,y)+∧(1−IR(y,z)−)),1−⋁y∈U(IR(x,y)−∧(1−IR(y,z)+))]=[⋀y∈U((1−IR(x,y)+)∨IR(y,z)−),⋀y∈U((1−IR(x,y)−)∨IR(y,z)+)]=⋀y∈U((1−IR(x,y))∨IR(y,z)).

Therefore (1) has been proven.

(1)⇒(3) First we prove that

(34) $$\begin{array}{c} {\bar{RIF}}_{\alpha }^{\prime }\left(A_{\alpha }\right)\subseteq {\underline{RIF}}_{\bar{1-\alpha }}^{\prime }\left({\bar{RIF}}_{\alpha }^{\prime }\left(A_{\alpha }\right)\right). \end{array}$$

Note that, for all *x* ∈ *U*, if $x\in {\bar{RIF}}_{\alpha }^{\prime }(A_{\alpha })$, then *IR*_*α*_(*x*)∩*A*_*α*_ ≠ *∅*; that is, there exists *z* ∈ *IR*_*α*_(*x*)∩*A*_*α*_. So we have *IR*(*x*, *z*) ≥ *α* and *A*(*z*) ≥ *α*.

Since

(35) α≤IR(x,z)≤⋀y∈U((1−IR(x,y))∨IR(y,z))=[⋀y∈U((1−IR(x,y)+)∨IR(y,z)−),⋀y∈U((1−IR(x,y)−)∨IR(y,z)+)],

we have

(36) $$\begin{array}{c} \overset{}{\underset{y\in U}{⋀}}\left(\left(1-IR{\left(x,y\right)}^{+}\right)\vee IR{\left(y,z\right)}^{-}\right)\geq \alpha^{-},\\ \overset{}{\underset{y\in U}{⋀}}\left(\left(1-IR{\left(x,y\right)}^{-}\right)\vee IR{\left(y,z\right)}^{+}\right)\geq \alpha^{+}. \end{array}$$

Hence, for all *y* ∈ *U*, *IR*(*x*, *y*)^+^ ≤ 1 − *α* ^−^ or *IR*(*y*, *z*)^−^ ≥ *α* ^−^and *IR*(*x*, *y*)^−^ ≤ 1 − *α* ^+^ or *IR*(*y*, *z*)^+^ ≥ *α* ^+^ imply that if *IR*(*x*, *y*)^+^ > 1 − *α* ^−^, then *IR*(*y*, *z*)^−^ ≥ *α* ^−^, and if *IR*(*x*, *y*)^−^ > 1 − *α* ^+^, then *IR*(*y*, *z*)^+^ ≥ *α* ^+^. It follows that *IR*(*x*, *y*)^+^ > 1 − *α* ^−^ and *IR*(*x*, *y*)^−^ > 1 − *α* ^+^ imply that *IR*(*y*, *z*)^−^ ≥ *α* ^−^ and *IR*(*y*, *z*)^+^ ≥ *α* ^+^; therefore, *IR*(*x*, *y*) > 1 − *α* implies that *IR*(*y*, *z*) ≥ *α*, because *IR*(*x*, *y*) > 1 − *α* and *IR*(*y*, *z*) ≥ *α* are equivalent to $y\in IR_{\bar{1-\alpha }}(x)$ and *z* ∈ *IR*_*α*_(*y*), respectively. If $y\in IR_{\bar{1-\alpha }}(x)$, then *z* ∈ *IR*_*α*_(*y*), and since *A*(*z*) ≥ *α*, $y\in IR_{\bar{1-\alpha }}(x)$ implies that *z* ∈ *IR*_*α*_(*y*)∩*A*_*α*_; that is, *IR*_*α*_(*y*)∩*A*_*α*_ ≠ *∅*. So $y\in IR_{\bar{1-\alpha }}(x)$ implies that $y\in {\bar{RIF}}_{\alpha }^{\prime }(A_{\alpha })$.

For arbitrary *y*, it follows that $IR_{\bar{1-\alpha }}(x)\subseteq {\bar{RIF}}_{\alpha }^{\prime }(A_{\alpha })$. Hence, $x\in {\underline{RIF}}_{\bar{1-\alpha }}^{\prime }\left({\bar{RIF}}_{\alpha }^{\prime }\left(A_{\alpha }\right)\right)$. Then, for arbitrary *x*, we obtain ${\bar{RIF}}_{\alpha }^{\prime }(A_{\alpha })\subseteq {\underline{RIF}}_{\bar{1-\alpha }}^{\prime }\left({\bar{RIF}}_{\alpha }^{\prime }\left(A_{\alpha }\right)\right)$.

By Lemma 37, it follows that

(37) RIF_′(RIF¯′(A))=⋃α∈[I]αRIF_1−α¯′((RIF¯′(A))α)⊇⋃α∈[I]αRIF_1−α¯′(RIF¯α′(Aα))⊇⋃α∈[I]αRIF¯α′(Aα)=RIF¯′(A).

Therefore ${\bar{RIF}}^{\prime }(A)\subseteq {\underline{RIF}}^{\prime }\left({\bar{RIF}}^{\prime }\left(A\right)\right)$.

(2)⇔(3) This conclusion follows immediately from the duality.

**Theorem 45 .**

Let (*U*, *IR*) be a generalized interval-valued fuzzy approximation space; then the following conditions are equivalent:

1. $\underset{}{\wedge_{y\in U}}((1-IR(x,y))\vee IR(y,x))=1,x\in U$;
2. *IR*(*x*, *y*) = 0  *or* *IR*(*y*, *x*) = 1, *x*, *y* ∈ *U*;
3. ${\bar{RIF}}^{\prime }({\underline{RIF}}^{\prime }(A))\subseteq A,A\in F^{I}(U)$;
4. $A\subseteq {\underline{RIF}}^{\prime }({\bar{RIF}}^{\prime }(A)),A\in F^{I}(U)$.

**Proof**

(1)⇔(2) We observe that, for all *x* ∈ *U*, $\underset{}{\wedge_{y\in U}}\left(\left(1-IR\left(x,y\right)\right)\vee IR\left(y,x\right)\right)=1$ if and only if ((1 − *IR*(*x*,*y*)^+^)∨*IR*(*y*,*x*)^−^) = 1 and ((1 − *IR*(*x*,*y*)^−^)∨*IR*(*y*,*x*)^+^) = 1; namely, for all *x* ∈ *U*, *IR*(*x*,*y*)^+^ = 0  or  *IR*(*y*,*x*)^−^ = 1 and *IR*(*x*,*y*)^−^ = 0 or *IR*(*y*,*x*)^+^ = 1.

On the one hand, if *IR*(*x*, *y*)^+^ = 0, then *IR*(*x*, *y*)^−^ = 0; we have *IR*(*x*, *y*) = 0. If *IR*(*x*,*y*)^+^ ≠ 0, then *IR*(*y*,*x*)^−^ = 1 and *IR*(*y*,*x*)^+^ = 1; we have *IR*(*y*, *x*) = 1.

Hence, $\underset{}{\wedge_{y\in U}}\left(\left(1-IR\left(x,y\right)\right)\vee IR\left(y,x\right)\right)=1$ if and only if for all *y* ∈ *U*, *IR*(*x*, *y*) = 0 or *IR*(*y*, *x*) = 1.

(2)⇒(4) We first prove that $A_{\alpha }\subseteq {\underline{RIF}}_{\bar{1-\alpha }}^{\prime }\left({\bar{RIF}}_{\alpha }^{\prime }\left(A_{\alpha }\right)\right)$.

Suppose that *x* ∈ *A*_*α*_, for all *y* ∈ *U*, *IR*(*x*, *y*) = 0 or *IR*(*y*, *x*) = 1 if and only if *IR*(*x*, *y*) ≠ 0 deduces *IR*(*y*, *x*) = 1. It follows that if *IR*(*x*, *y*) > 1 − *α*, then *IR*(*y*, *x*) ≥ *α*; that is, *x* ∈ *IR*_*α*_(*y*). Further since *x* ∈ *A*_*α*_, *IR*(*x*, *y*) > 1 − *α* implies that *x* ∈ *IR*_*α*_(*y*)∩*A*_*α*_. So *IR*_*α*_(*y*)∩*A*_*α*_ ≠ *∅*. Note that *IR*(*x*, *y*) > 1 − *α* and *IR*_*α*_(*y*)∩*A*_*α*_ ≠ *∅* are equivalent to $y\in IR_{\bar{1-\alpha }}(x)$ and $y\in {\bar{RIF}}_{\alpha }^{\prime }(A_{\alpha })$, respectively; we have if *IR*(*x*, *y*) > 1 − *α*, then *x* ∈ *IR*_*α*_(*y*)∩*A*_*α*_. It shows that if $y\in IR_{\bar{1-\alpha }}(x)$, then $y\in {\bar{RIF}}_{\alpha }^{\prime }(A_{\alpha })$. By the arbitrary *y*, $IR_{\bar{1-\alpha }}(x)\subseteq {\bar{RIF}}_{\alpha }^{\prime }(A_{\alpha })$ holds; namely, $x\in {\underline{RIF}}_{\bar{1-\alpha }}^{\prime }({\bar{RIF}}_{\alpha }^{\prime }(A_{\alpha }))$. For any *x*, $A_{\alpha }\in {\underline{RIF}}_{\bar{1-\alpha }}^{\prime }\left({\bar{RIF}}_{\alpha }^{\prime }\left(A_{\alpha }\right)\right)$ holds.

On the other hand, in view of Lemma 37, we have

(38) RIF_′(RIF¯′(A))=⋃α∈[I]αRIF_1−α¯′((RIF¯′(A))α)⊇⋃α∈[I]αRIF_1−α¯′(RIF¯α′(Aα))⊇⋃α∈[I]αAα=A.

(3)⇒(1) For all *x*, *z* ∈ *U*, from the proof of “(2)⇒(1)” in Theorem 44, we know that $\underset{}{\vee_{y\in U}}\left(IR\left(x,y\right)\wedge \left(1-IR\left(y,z\right)\right)\right)={\bar{RIF}}^{\prime }({\underline{RIF}}^{\prime }(1_{U/\{x\}}))(z)\;$.

Furthermore, since ${\bar{RIF}}^{\prime }({\underline{RIF}}^{\prime }(1_{U/\{x\}}))(z)\leq 1_{U/\{x\}}(z)$, it follows that $\underset{}{\vee_{y\in U}}(IR(x,y)\wedge (1-IR(y,z)))\leq 1_{U/\{x\}}(z)$; namely, $\underset{}{\wedge_{y\in U}}((1-IR(x,y))\vee IR(y,z))\geq 1-1_{U/\{x\}}(z)$.

When *z* = *x*, we have $\underset{}{\wedge_{y\in U}}((1-IR(x,y))\vee IR(y,x))\geq 1$. Because the value of $\underset{}{\wedge_{y\in U}}((1-IR(x,y))\vee IR(y,x))$ is restricted in [*I*], we have $\underset{}{\wedge_{y\in U}}((1-IR(x,y))\vee IR(y,x))=1$.

(3)⇔(4) This conclusion follows immediately from the duality.

**Theorem 46 .**

Let (*U*, *IR*) be a generalized interval-valued fuzzy approximation space.

1. If *IR* is reflexive and transitive, then ${\underline{RIF}}^{\prime }(A)={\underline{RIF}}^{\prime }\left({\underline{RIF}}^{\prime }\left(A\right)\right)$ and ${\bar{RIF}}^{\prime }(A)={\bar{RIF}}^{\prime }\left({\bar{RIF}}^{\prime }\left(A\right)\right)$, for all *A* ∈ *F* ^*I*^(*U*).
2. If *IR* is reflexive and $IR\left(x,z\right)\leq \underset{}{\wedge_{y\in U}}\left(\left(1-IR\left(x,y\right)\right)\vee IR\left(y,z\right)\right)$, for all *x*, *z* ∈ *U*, then ${\underline{RIF}}^{\prime }\left(A\right)={\bar{RIF}}^{\prime }\left({\underline{RIF}}^{\prime }\left(A\right)\right)\;$and ${\bar{RIF}}^{\prime }(A)={\underline{RIF}}^{\prime }({\bar{RIF}}^{\prime }(A))$, for all *A* ∈ *F* ^*I*^(*U*).

**Proof**

Theorem 46 is proved easily by Theorems 34, 41, and 44.

According to duality and Theorem 46, one can obtain the next corollary.

**Corollary 47 .**

Suppose that (*U*, *IR*) is a generalized interval-valued fuzzy approximation space.

(1) If *IR* is reflexive and transitive, then
  (39) ~RIF_′(A)=RIF¯′(~RIF_′(A))=RIF¯′(RIF¯′(~A)),∀A∈FI(U),~RIF¯′(A)=RIF_′(~RIF¯′(A))=RIF_′(RIF_′(~A)),∀A∈FI(U).
(2) If *IR* is reflexive and $IR(x,z)\leq \underset{}{\wedge_{y\in U}}\left(\left(1-IR\left(x,y\right)\right)\vee IR\left(y,z\right)\right)$, for all *x*, *z* ∈ *U*, then
  (40) ~RIF_′(A)=RIF_′(~RIF_′(A))=RIF_′(RIF¯′(~A)),∀A∈FI(U),~RIF¯′(A)=RIF¯′(~RIF¯′(A))=RIF¯′(RIF_′(~A)),∀A∈FI(U).

## 6. Conclusion and Future Work

In this paper, we proposed two types of the generalized interval-valued fuzzy approximation operators by integrating the generalized rough set theory and interval-valued fuzzy sets as well as fuzzy relations. The equivalence of these two types of the generalized interval-valued fuzzy approximation operators has been examined. Furthermore, we also demonstrated the duality of the lower and upper generalized interval-valued fuzzy approximation operators and discussed the properties of the generalized interval-valued fuzzy approximation operators under different interval-valued fuzzy relations.

In this paper, one can prove that the binary relation obtained by calculating *α*-cut set or strong *α*-cut set to an interval-valued fuzzy relation, for all *α* ∈ [*I*], still satisfies the corresponding definition of Definition 11 under the classical binary relation; that is, if* IR* is reflexive, symmetric, and transitive, respectively, then *IR*(*IR* *a*) is reflexive, symmetric, and transitive, respectively, under the classical binary relation. Thus, if *IR* can satisfy the above functions, this technology can be applied in reasoning, learning, and decision-making. In Sections 4 and 5, the definitions and theorems provide some theoretical bases for reasoning, learning, and decision-making.
